# Thermodynamic, Kinetic, and Equilibrium Parameters for the Removal of Lead and Cadmium from Aqueous Solutions with Calcium Alginate Beads

**DOI:** 10.1155/2014/647512

**Published:** 2014-01-27

**Authors:** Ruth Alfaro-Cuevas-Villanueva, Aura Roxana Hidalgo-Vázquez, Consuelo de Jesús Cortés Penagos, Raúl Cortés-Martínez

**Affiliations:** ^1^Instituto de Investigaciones Químico Biológicas, Universidad Michoacana de San Nicolás de Hidalgo, CP 58060, Edif. B1., CU, Morelia, MICH, Mexico; ^2^Facultad de Químico Farmacobiología, Universidad Michoacana de San Nicolás de Hidalgo, Tzintzuntzan 173 Col. Matamoros, CP 58240, Morelia, MICH, Mexico

## Abstract

The sorption of cadmium (Cd) and lead (Pb) by calcium alginate beads (CAB) from aqueous solutions in batch systems was investigated. The kinetic and thermodynamic parameters, as well as the sorption capacities of CAB in each system at different temperatures, were evaluated. The rate of sorption for both metals was rapid in the first 10 minutes and reached a maximum in 50 minutes. Sorption kinetic data were fitted to Lagergren, pseudo-second-order and Elovich models and it was found that the second-order kinetic model describes these data for the two metals; comparing kinetic parameters for Cd and Pb sorption a higher kinetic rate (*K*_2_) for Pb was observed, indicating that the interaction between lead cations and alginate beads was faster than for cadmium. Similarly, isotherm data were fitted to different models reported in literature and it was found that the Langmuir-Freundlich (L-F) and Dubinin-Radushkevich (D-R) models describe the isotherms in all cases. CAB sorption capacity for cadmium was 27.4 mg/g and 150.4 mg/g for lead, at 25°C. Sorption capacities of Cd and Pb increase as temperature rises. According to the thermodynamic parameters, the cadmium and lead adsorption process was spontaneous and endothermic. It was also found that pH has an important effect on the adsorption of these metals by CAB, as more were removed at pH values between 6 and 7.

## 1. Introduction

Water, air, and soil pollution by heavy metals is one of the most serious environmental problems and is very difficult to solve [1]. Heavy metal contamination exists in wastewater streams from different industries such as electroplating, mining, and tanneries, among others. Some of the metals associated with these activities are cadmium, copper, chromium, iron, mercury, nickel, lead, and zinc [2]. These metals are not biodegradable, and they tend to accumulate in living organisms, causing diseases and disorders. For these reasons, they are considered priority pollutants. Despite the negative effects, these elements are often discharged into the environment and reach concentrations above those permitted by law [3].

Heavy metals, such as lead (Pb) and cadmium (Cd), are a sanitary and ecological threat. They are highly toxic and recalcitrant even at very low concentrations, and they can pollute drinking water resources. Therefore, research is important to fully understand the systems and technologies needed for their removal [4].

Traditional methods for removing heavy metals, including chemical precipitation and filtration, redox reactions, electrochemical treatments, reverse osmosis, ion exchange, adsorption, and evaporation [4], generally have several disadvantages, such as incomplete metal removal, expensive equipment and monitoring system requirements, high reagent or energy requirements and the generation of toxic sludge, or other waste products that require disposal. Further, they may be ineffective or extremely expensive when metals' concentrations in wastewater are in the range of 10–100 mg/L [5, 6]. Therefore, it is necessary to find simple and consistent technologies that use local resources to remove metals at a low cost [7].

Among these technologies, adsorption appears to be the most effective to control heavy metal pollution in wastewaters since it is a relatively simple technology used to remove ions from aqueous solutions. Many studies have reported the possible utilization of conventional adsorbents like activated carbon, zeolites, clays, and so forth [8, 9]. In recent years, research about the use of nanoparticles for the removal of metal ions from wastewaters has increased due to the high adsorption capacities, low water solubility, availability, low cost, and high stability under oxidizing and reducing conditions of these materials. Materials like carbon nanoparticles, magnetic hydroxyapatite nanoparticles, nickel oxide nanoparticles, titanium dioxide, and so forth have been used to remove cadmium and lead from aqueous solutions [10].

Biosorption is an alternative to established methods that, in recent years, has proven its high efficiency for removing heavy metals from wastewater. This method involves the removal of metals through physical phenomena, such as adsorption, ion exchange or metabolic processes, by using living or dead biomass as an adsorbent with chemically active sites or functional groups. In addition, biosorption is a precise and selective method that requires only a few minutes of treatment [11]. Recently, low cost biosorbents have been prepared from different wastes, such as peanut shells, corn cobs, rice hulls, sawdust, sugarcane bagasse, coffee and orange wastes, and yeast, obtained from the timber, fishing, and agricultural industries [12–17]. These biomaterials have been recognized as potential alternatives to conventional technologies for removing metals from wastewater. Therefore, the investigation of this type of technology is important, and its development could be especially competitive in the treatment of industrial effluents by allowing the recovery of metals and the reuse of biomass and water.

The use of dead biomass has some advantages over the use of living biomass: it is not necessary to add nutrients, the adsorbent is immune to toxicity or adverse operating conditions, the processes are not governed by biological constraints, the recovery of metals is easy, and biomass can act in ion exchange. However, the use of dead biomass has some disadvantages such as rapid saturation of the solid, high sensitivity to changes in pH and the fact that the oxidation state of metal cannot be altered biologically [18].

One of the promising techniques for adsorbing metals is the use of biopolymers and non-living organisms as sorbents. Because biopolymers and non-living organisms have several functional groups, they are expected to display different affinities to various metal ions. Biopolymers are extracted from non-living organisms and have common chemical properties with them. Alginate is a component of the outer cell wall of brown algae. The commercial alginate exists in a powder form as sodium alginate, and it can form a viscous solution when it is dissolved in water. It is usually prepared as calcium alginate when it is used for metal ion removal. The major component of the alginate is alginic acid, which is a polymer composed of unbranched chains of 1,4-linked *β*-D-mannuronic and *α*-L-guluronic acids. They are rich in carboxyl groups, which are the main functional groups involved in heavy metal biosorption [4]. The potential binding sites in biopolymers and non-living organisms are carboxylate, amine, phosphate, sulfate, hydroxyl, and other chemical functional groups. Through surface charge studies, it was found that the availability of the free active sites depends on pH. With an increase in pH, the charged sites on the surface of calcium alginate become more negative. As a result, the uptake of metal ions increases with an increase in pH [19].

The aim of this study was to evaluate the removal of lead and cadmium using calcium alginate beads as biosorbents by obtaining the kinetics and equilibrium parameters of each metal-biosorbent system, as well as to determine the influence of pH and temperature on the processes in batch systems.

## 2. Materials and Methods

### 2.1. Preparation and Characterization of Alginate Beads

The alginate spheres were prepared using sodium alginate as a precursor after a 2% (w/v) sodium alginate solution was prepared with deionized water. Subsequently, the solution was placed on a hot stirring plate for 3 h at 50°C until a transparent and viscous solution was observed, and this solution was allowed to stand for 24 h. Additionally, 500 mL of 150 mM calcium chloride solution was prepared. For the preparation of the alginate spheres, sodium alginate solution was added dropwise at approximately one drop per second to a beaker containing the calcium chloride solution, and it was stirred gently at approximately 60 rpm to allow the spheres to form. The spheres that were formed were left stirring for 24 h. Then, the spheres were separated from the solution and washed 5 times with deionized water to produce deprotonated alginate spheres. Finally, the spheres were dried in an oven at 35°C. The calcium alginate beads (CAB) obtained in this procedure were sieved to select particles of approximately 1 mm which were then used in the biosorption experiments.

### 2.2. Kinetics of Cadmium and Lead Biosorption

To determine the kinetics of Cd and Pb biosorption, batch-type experiments with alginate beads and aqueous solutions of lead and cadmium were conducted. For this purpose, 0.5 g of CAB was placed in centrifuge tubes with 5 mL of 0.001 N Pb(NO_3_)_2_ or Cd(NO_3_)_2_·4H_2_O solutions. The tubes were stirred at room temperature (25°C) for contact times ranging from 5 to 180 min. After each contact time was reached, the solution was filtered and the supernatant was placed in vials for later analysis of lead and cadmium by atomic absorption spectroscopy (AAS). The same procedure was used to determine the kinetics of biosorption of lead and cadmium onto CAB at 35 and 50°C. The quantities of cadmium and lead adsorbed onto CAB were calculated from the initial concentration of the solution

(1) $$\begin{array}{c} q=\frac{V\left(C_{0}-C_{e}\right)}{M}, \end{array}$$

where *q* is the measured sorption per unit weight of solid, *V* is the volume of solution, *C*_0_ and *C*_*e*_ are the initial and equilibrium concentrations of metal, respectively, and *M* is the dry weight of biosorbent. All sorption experiments were performed three times to ascertain the reproducibility of the results, and the mean values were considered. Blank experiments showed no detectable lead or cadmium adsorbed onto the walls of the centrifuge tubes.

### 2.3. Influence of pH

To establish the pH value at which CAB removes the most metal and to determine the influence of this parameter on the biosorbent material, batch-type adsorption experiments with CAB and 0.001 N aqueous solutions of cadmium and lead were performed. Different solutions with pH values between 3 and 9 were prepared to determine the influence of this parameter on the biosorption process, and this was set as a parameter to carry out further experiments. These values were adjusted by adding small amounts of 0.1 M solutions of HCl or NaOH, as appropriate. Centrifuge tubes were filled with 0.5 g of CAB and 5 mL of aqueous solutions of cadmium and lead, separately, at different pH values. The tubes were placed in a thermostat-adjusted water bath agitator at 25°C for the equilibrium time, as determined by the kinetics of biosorption. At the end of contact time, the solution was filtered, the supernatant was placed in vials, and it was analyzed for Cd and Pb by AAS, as mentioned above. The tests to determine the influence of pH were performed in triplicate.

### 2.4. Biosorption Isotherms

Batch type experiments were carried out with CAB and aqueous solutions of lead and cadmium at concentrations from 0.001 N to 0.5 N and at temperatures of 25, 35, and 50°C to determine the effect of temperature on the maximum biosorption capacities of alginate for these metals. In centrifuge tubes, 0.5 g of CAB samples was put into contact with 5 mL of cadmium and lead aqueous, separately, at different concentrations. The tubes were placed in a thermostat-adjusted water bath agitator at different temperatures for the equilibrium time determined by the biosorption kinetics experiments. At the end of the contact time, the solution was filtered, and the supernatant was placed in vials for metal analysis by AAS, as mentioned above. The experiments were performed in triplicate, as in the previous cases.

## 3. Results and Discussion

### 3.1. Kinetics of Biosorption of Cadmium and Lead

**Figure 1:**
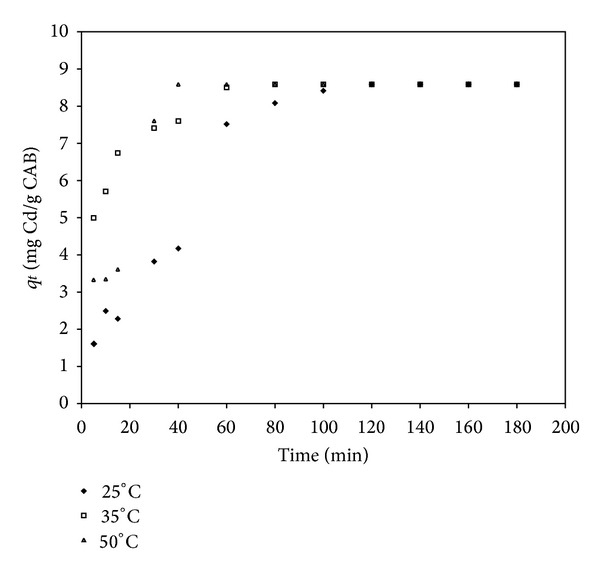
Adsorption capacity (*q*_*t*_) of cadmium by CAB at different temperatures (°C) versus time (min).

**Figure 2:**
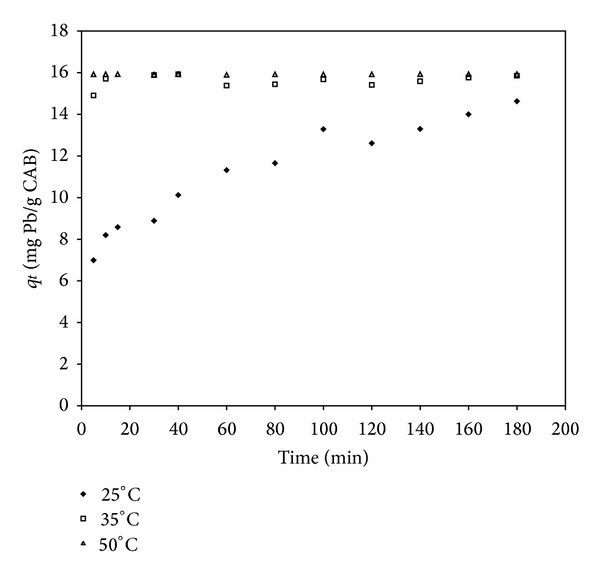
Adsorption capacity (*q*_*t*_) of lead by CAB at different temperatures (°C) versus time (min).

Figures 1 and 2 show the results obtained from the tests of cadmium and lead removal by CAB at different temperatures and as a function of time, respectively. The curves of cadmium removal are characterized by a relatively fast sorption in the first 60 minutes of contact time at all temperatures (Figure 1). During the first two hours of contact time, 8.5 mg/g of the sorption occurred, and the equilibrium was reached. A total cadmium removal from the aqueous solution, near 100%, was observed at 50°C. In the case of lead removal by CAB in the same experimental conditions, the kinetic sorption behavior was different to Figure 2 since maximum adsorption of Pb is reached in the first minutes of reaction at 35 and 50°C. Additionally, at 25°C the maximum adsorption of Pb was obtained after 3 h. During the first two hours of contact time, 15.9 mg/g of lead sorption occurred, and the equilibrium was reached. A total removal of the lead from the aqueous solution, near 100%, was observed at 35 and 50°C. From these data, it can be observed that temperature plays an important role in the kinetics of cadmium and lead removal by this biosorbent.

Experimental data were fit to empirical kinetic models by nonlinear regression analyses to obtain the kinetic parameters for each of the systems studied at different temperatures. Lagergren's pseudo-first-order model, a pseudo-second-order model, and the Elovich equation were used to fit the data. Pseudo-first-order and pseudo-second-order equations can be used while assuming that the measured concentrations are equal to surface concentrations. These models are expressed as follows.

  Lagergren's pseudo-first-order model (2) [20]:

(2) $$\begin{array}{c} q_{t}=q_{e}\left(1-e^{-K_{L^{t}}}\right); \end{array}$$

 pseudo-second-order model (3) [21]:

(3) $$\begin{array}{c} \frac{t}{q_{t}}=\frac{1}{K_{2}qe^{2}}+\frac{t}{qe}; \end{array}$$

**Figure 3:**
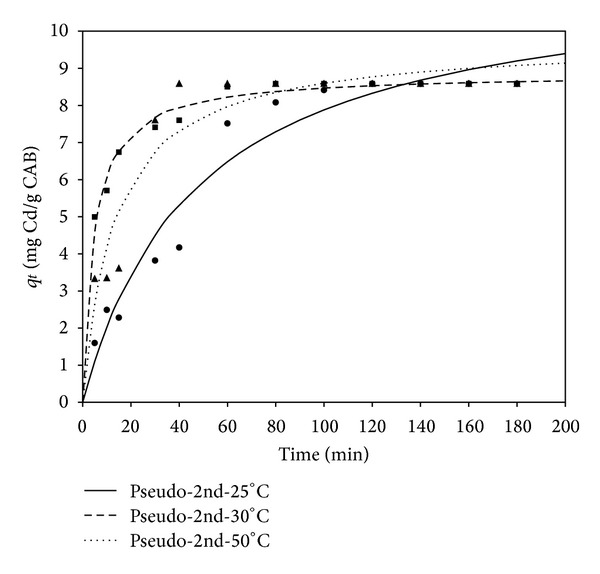
Adsorption capacity (*q*_*t*_) of cadmium by CAB at different temperatures (°C) versus time (min), adjusted to a pseudo-second-order model.

**Figure 4:**
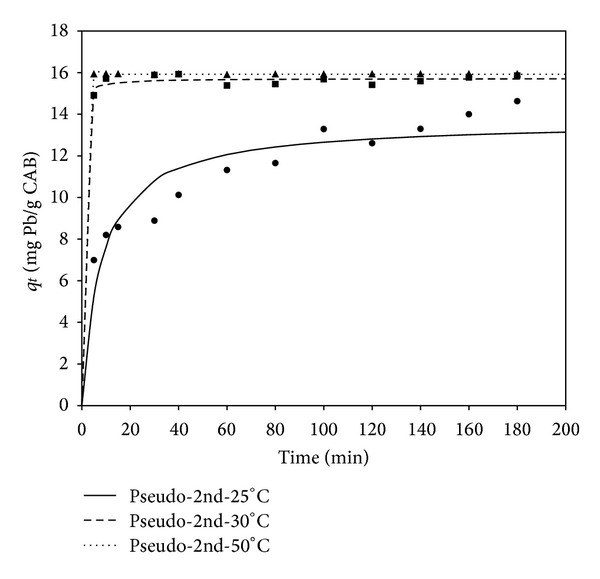
Adsorption capacity (*q*_*t*_) of lead by CAB at different temperatures (°C) versus time (min), adjusted to a pseudo-second-order model.

 Elovich equation (4) [22, 23]:

(4) $$\begin{array}{c} q_{t}=\frac{1}{b}\ln\left(1+abt\right), \end{array}$$

where *q*_*t*_ is the metal concentration at time, *t*, per weight of adsorbent (mg/g), *q*_*e*_ is the concentration of metal removed at equilibrium per weight of adsorbent (mg/g), *K*_*L*_ is the pseudo-first-order kinetic constant (min^−1^), *K*_2_ is the pseudo-second-order rate constant of sorption (g/mg·h), and *a* and *b* are Elovich constants related to the initial adsorption rate (mg/g·h) and the desorption rate (g/mg), respectively. In general, the experimental data for the sorption of cadmium and lead on CAB, as a function of time, were best fitted to the pseudo-second-order model (Figures 3 and 4), with correlation coefficients (*R*) between 0.918 and 0.999.

The kinetic parameters of the models are shown in Tables 1 and 2 for cadmium and lead, respectively, at different temperatures. The low correlation coefficient values obtained for the pseudo-first-order model indicate that sorption is not occurring exclusively onto one site per ion [24]. In accordance with the pseudo second-order reaction mechanism, the overall rate of Cd sorption processes appears to be controlled by chemical processes, through sharing of electrons between biosorbent and sorbate, or covalent forces, through the exchange of electrons between the particles involved [25, 26]. For the pseudo-second-order constant (*K*_2_), it can be observed that this parameter increases as temperature rises for Cd and Pb sorption kinetics, thereby implying that the biosorption system reached equilibrium faster at higher temperatures. Additionally, the sorption processes could be enhanced by an increment in temperature, which suggests an endothermic nature of the Cd and Pb biosorption processes. This behavior could be attributed to an increase in the probability of the collision between active surface binding sites and the sorbate and a decrease in the thickness of the boundary layer surrounding the biosorbent at high temperatures. Thus, the mass transfer resistance of sorbate in the boundary layer decreased [27]. The correlation coefficients obtained were greater than 0.98, and the adequate fitting of theoretical and experimental *q*_*e*_ values at all temperatures suggests the applicability of these models, based on the assumption that the rate-limiting step may be chemisorption, in explaining the kinetics of biosorption for the entire sorption period. This indicated that the pseudo-second-order kinetic model describes the cadmium and lead biosorption kinetics adequately. Comparing kinetic parameters for Cd and Pb biosorption, lead showed a higher kinetic rate (*K*_2_), indicating that the interaction between lead cations and alginate beads was faster compared to the interaction with cadmium cations.

### 3.2. The Influence of pH on Cd and Pb Biosorption

**Figure 5:**
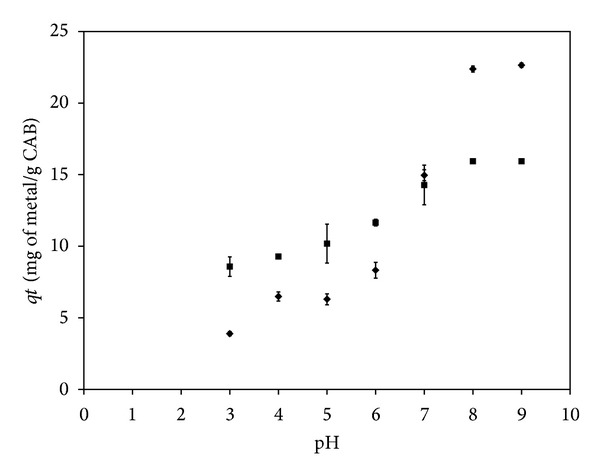
Influence of pH on the biosorption of Cd (◆) and Pb (■) by CAB.

The pH is an important parameter to be considered in sorption processes because it may affect both the properties of the adsorbent and the composition of the solution. It is also important because of the ionization of surface functional groups and the composition of solutions [28].  Figure 5 shows the variation in Cd and Pb sorption capacities at various pH values for the calcium alginate beads and a similar behavior for both metals. The removal of Cd and Pb was the highest in basic conditions, but this increase could be explained by metal precipitation due to formation of metal hydroxides at these pH values [29–31]. At highly acidic conditions, Cd and Pb removal showed a lower metal uptake; this behavior could be attributed to competition for metal binding sites between metal ions and hydrogen (H^+^) and hydronium (H_3_O^+^) ions because it has been established that the hydrogen ion is a strong competing adsorbate [31, 32]. Moreover, the p*K*_*a*_ values for the carboxylic groups of the *α*-L-guluronic acid and *β*-D-mannuronic acid of the alginate have been reported to be in the range of 3.4 to 3.9 [33], which indicates a positive charge of the biosorbent below these values that results in a decrease of metal biosorption. As the pH of the medium rises, this competitive effect is minimized and the solubility of Pb or Cd decreases and it favors the formation of hydrolyzed species with a larger ion that facilitates contact between the functional groups and the metal [34, 35]. Based on these facts, it can be deemed that the optimum pH values for Cd and Pb biosorption by CAB were between 6 and 7, as it is observed in Figure 5.

### 3.3. Cadmium and Lead Biosorption Isotherms

**Figure 6:**
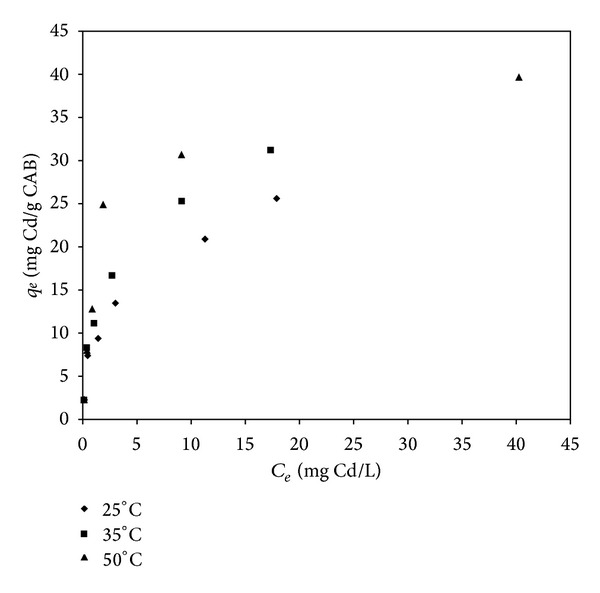
Isotherms of cadmium biosorption by CAB at 25°C, 35°C, and 50°C.

**Figure 7:**
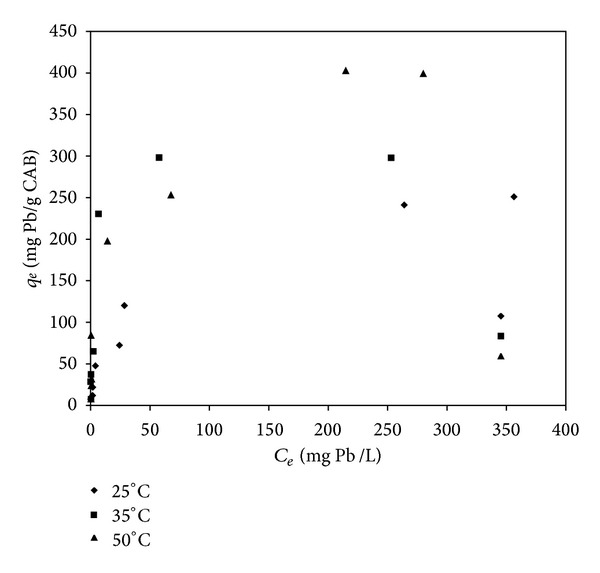
Isotherms of lead biosorption by CAB at 25°C, 35°C, and 50°C.

The sorption isotherms of cadmium and lead biosorption using CAB at different temperatures are shown in Figures 6 and 7, respectively. The gradual decrease in adsorption rate of Pb and Cd with an increasing initial concentration of these metals in the solution shows the continued saturation of the available binding sites.

According to the results shown in Figure 6, it can be observed that the amount of Cd adsorbed by CAB increased by raising the initial metal concentration, and it followed a nonlinear-type isotherm. The maximum amount of Cd adsorbed by CAB increased slightly with an increase in temperature. This behavior could be due to the surface binding reactions that occur simultaneously with changes in temperature [32] and it indicates that calcium alginate beads have a greater affinity for Cd when the temperature increases (Figure 6). It was reported that cadmium biosorption by different types of biosorbents was weakly affected by temperature in the test range of 20°C to 50°C [27, 32]. In the case of lead biosorption by CAB (Figure 7), the results showed a similar behavior when compared with the cadmium isotherms, but in this case, a significant increase in sorption capacity was observed.

Experimental data from these plots were fitted to the following isotherm models by nonlinear regression analysis ((5)–(7)):

 Freundlich:

(5) $$\begin{array}{c} q_{e}=K_{F}C_{e}^{1/n_{F}}, \end{array}$$

where *q*_*e*_ is the amount of solute per unit weight of adsorbent (mg/g), *C*_*e*_ is the solute concentration in the solution at equilibrium (mg/L), *K*_*F*_ is the equilibrium constant indicative of adsorption capacity, and *n*_*F*_ is the adsorption equilibrium constant whose reciprocal is indicative of adsorption intensity,

 Langmuir:

(6) $$\begin{array}{c} q_{e}=\frac{q_{0}a_{L}C_{e}}{1+a_{L}C_{e}}=\frac{K_{L}C_{e}}{1+a_{L}C_{e}}, \end{array}$$

where *q*_*e*_ is the amount of solute per unit weight of adsorbent (mg/g), *C*_*e*_ is the solute concentration in the solution at equilibrium (mg/L), *q*_0_ is the amount of solute retained per unit weight of adsorbent in forming a complete monolayer on the surface (mg/g), *a*_*L*_ is the constant related to the energy or net enthalpy of adsorption, and *K*_*L*_ is the Langmuir constant, (L/g). Langmuir-Freundlich model can be expressed as follows:

(7) $$\begin{array}{c} q_{e}=\frac{K_{LF}C_{e}^{n}}{1+a_{LF}C_{e}^{n}}, \end{array}$$

where *q*_*e*_ is the amount of solute per unit weight of adsorbent (mg/g), *C*_*e*_ is the solute concentration in the solution at equilibrium (mg/L), and *K*_*LF*_ and *a*_*LF*_ are empirical constants.

The isothermal biosorption parameters for these models are shown in Tables 3 and 4 for cadmium and lead, respectively. For cadmium (Table 3), the experimental data were best fit by the Freundlich isotherm model. Although the Freundlich model is often considered an empirical model, it is widely accepted that this model describes adsorption with heterogeneous energy [36]. Therefore, it is assumed that heterogeneous biosorption plays an important role in the removal of cadmium and lead by CAB. It can also be observed that maximum biosorption capacities increase at higher temperatures, suggesting an endothermic nature of the cadmium sorption process. Moreover, the Freundlich parameter *n* indicates the favorability of the adsorption. An adsorption intensity of *n* greater than 1 is favorable at high concentrations but not at lower concentrations. Values of this parameter are higher than 1 for all temperatures and this indicates a sorption intensity that is favorable at high concentrations and at high temperatures.

All isotherm data for lead (Table 4) were best adjusted by the Langmuir-Freundlich model, and the correlation coefficients (*R*) obtained by nonlinear regression analyses ranged from 0.986 to 0.991. The Freundlich and Langmuir models tested to fit the equilibrium experimental data showed significantly lower *R* values in all cases. At low adsorbate concentrations, the Langmuir-Freundlich isotherm (7) effectively reduces to the Freundlich isotherm, and thus it does not obey Henry's Law. At high sorbate concentrations, this isotherm predicts a monolayer sorption capacity characteristic of the Langmuir isotherm [37]. The Langmuir-Freundlich model has been widely used to describe data from the equilibrium of adsorption onto heterogeneous surfaces. Thus, the fact that the lead equilibrium data fits well within this model suggests that Pb biosorption onto CAB at different temperatures is of a heterogeneous nature. Similar results have been reported for lead sorption by different types of biosorbents [14, 33].

### 3.4. Thermodynamic Parameters

In this research, the thermodynamic parameters, such as the enthalpy change (Δ*H*) and the entropy change (Δ*S*) when CAB is an adsorbent for cadmium and lead, were calculated as follows ((8)–(9)) [38]:

(8) $$\begin{array}{c} K_{c}=\frac{C_{\text{ad}}}{C_{e}}. \end{array}$$

If *F*_*e*_ is the fractional conversion of the sorption at equilibrium, then metal concentrations in the adsorbent and the solution can be expressed as follows:

(9) $$\begin{array}{c} C_{e}=C_{i}\left(1-F_{e}\right),\\ C_{\text{ad}}=C_{i}F_{e}, \end{array}$$

where *K*_*c*_ is the equilibrium constant, *C*_*e*_ is the concentration of the metal in the solution at equilibrium (mg/L), *C*_*i*_ is the initial concentration of the metal in the solution at equilibrium (mg/L), and *C*_ad_ is the concentration of the metal in the adsorbent at equilibrium (mg/L). Equations (9) can be rearranged to produce

(10) $$\begin{array}{c} K_{c}=\frac{F_{e}}{1-F_{e}}. \end{array}$$

Equation (10) shows that the equilibrium constant is independent of the quantity of adsorbent and the volume of solution. The equilibrium constants (*K*_*c*_) were calculated at different temperatures for both of the metals adsorbed by CAB. The thermodynamic parameters were calculated using these values and van't Hoff equation (11) [38, 39]:

(11) $$\begin{array}{c} \ln K_{c}=\frac{\Delta S}{R}-\frac{\Delta H}{RT}, \end{array}$$

where *K*_*c*_ is the equilibrium constant, Δ*S* is the change in the entropy of the process, Δ*H* is the change in the enthalpy of the process, and *T* is the temperature (K).

The values of Δ*H* and Δ*S* were calculated from the slopes and intercepts of the plots of ln⁡*K*_*c*_ as a function of 1/*T* for Cd and Pb (not shown). Values of Δ*H* = 175.91 J/mol and Δ*S* = 3.3 J/mol K were obtained for cadmium, and values of Δ*H* = 188.9 J/mol and Δ*S* = 4.36 J/mol K were obtained for lead. Positive values of Δ*H* suggest endothermic sorption of metals on CAB, and the positive value of Δ*S* confirms the increase in randomness at the solid-solution interface during biosorption.

The experimental data were adjusted to the model of Dubinin-Radushkevich (D-R) to determine if the ion exchange process is determinant in the sorption process [39]. The D-R isotherm [40] assumes a heterogeneous surface

(12) $$\begin{array}{c} q_{e}=X_{m}\exp\left(-K\varepsilon^{2}\right), \end{array}$$

where *ε* (the Polanyi potential) = *RT*ln⁡(1 + 1/*C*_*e*_), *q*_*e*_ is the amount of metal ions adsorbed per unit weight of CAB (mg/g), *X*_*m*_ the adsorption capacity of the sorbent (mg/g), *C*_*e*_ is the equilibrium concentration of metal ions in solution (mg/L), *K* is a constant related to the adsorption energy (mol^2^/kJ^2^), *R* the gas constant (kJ/K mol), and *T*is the temperature (K). The D-R isotherm can be expressed in linear form [41]:

(13) $$\begin{array}{c} \ln q_{e}=\ln X_{m}-K\varepsilon^{2}. \end{array}$$

The values of *X*_*m*_ and *K* were calculated from the slopes and intercepts of the plots of ln⁡*q*_*e*_ as a function of *ε* ^2^ for Cd and lead biosorption onto CAB. The energy of sorption, which is defined as the change of energy when one mole of the ion is transferred to the surface of the solid, can be calculated using the value of *K* [42]:

(14) $$\begin{array}{c} \Delta G=-E={\left(-2k\right)}^{-1/2}. \end{array}$$

The magnitude of *E* can be related to the reaction mechanism. If the value of *E* is between 8 and 16 kJ/mol, ion exchange is the main sorption process in the system. If the value is lower than 8 kJ/mol, physical sorption is the main sorption mechanism [42].

The regression parameters and correlation coefficients (*R* ^2^) presented in Tables 3, 4, and 5 indicate that the adsorption data were best fitted by the Langmuir-Freundlich adsorption isotherm for both metals. However, under industrial conditions, the mixing of the adsorbent and the wastewater solution would be imperfect. Thus, the adsorbent surface is less likely to be homogeneous, and the assumptions required by the Langmuir isotherm would be less likely to be correct [41]. The good fit of the D-R isotherm, with all *R* ^2^ values greater than 0.9, suggests that these isotherms could be more appropriate under industrial conditions. For instance, at 25°C, the values of the adsorption capacity (*q*_0_) in the Langmuir isotherm were 27.4, 32.9, and 39.3 mg/g for Cd at 25, 35, and 50°C, respectively, and the corresponding values of adsorption capacity (*X*_*m*_) in the D-R isotherm were 20.4, 21.7, and 26.6 mg/g at these temperatures. The adsorption energies (*E*) are less than 8 kJ/mol for all cases at different temperatures (Table 5), except for Pb at 50°C, suggesting that physical forces at all studied temperatures dominated the sorption process and that these forces were more important than ion exchange and particle diffusion [41]. These parameters (Table 5) also show that the adsorption process is spontaneous and that the degree of spontaneity of the reaction increases with increasing temperature. As mentioned above, the overall adsorption process seems to be endothermic. This result also supports the suggestion that the adsorption capacity of CAB for all metals increases with increasing temperature. Additionally, the Δ*S* values were positive, which means that entropy increases as a result of adsorption. This occurs as a result of a redistribution of energy between the adsorbate and the adsorbent. Before adsorption occurs, the heavy metal ions near the surface of the adsorbent will be more ordered than in the subsequent adsorbed state, and the ratio of free heavy metal ions to ions interacting with the adsorbent will be higher compared to the adsorbed state. As a result, the distribution of rotational and translational energy among a small number of molecules will increase with increasing adsorption by producing a positive value of Δ*S*, and randomness will increase at the solid-solution interface during the process of adsorption. Thus, adsorption is likely to occur spontaneously at normal and high temperatures because Δ*H* is greater than 0 and Δ*S* is greater than 0 [41].

## 4. Conclusions

In general, it can be concluded that calcium alginate has good properties for the sorption of cadmium and lead from aqueous solutions. The pseudo-second-order model describes the cadmium and lead sorption kinetics using calcium alginate beads (CAB) at different temperature. The Langmuir-Freundlich model best describes the isotherm's experimental data. The sorption temperature is an important parameter that affects the sorption for cadmium and lead on CAB, and the sorption of both metals increases as the temperature increases, which indicates that the sorption process is endothermic. Thermodynamic parameters showed that physical forces at all studied temperatures dominated the sorption process, the adsorption process is spontaneous, and the degree of spontaneity of the reaction increases with increasing temperature. Finally, the calcium alginate beads had characteristics that were better for the removal of lead than for the removal of cadmium.

## Figures and Tables

**Table 1:** Kinetic parameters of cadmium sorption by calcium alginate beads at different temperatures.

Temperature (°C)	Pseudo-first-order (Lagergren)			Pseudo-second-order			Elovich		
	*K*_*L*_ (min^−1^)	*q*_*e*_ (mg/g)	*R*	*K*_2_ (min^−1^)	*q*_*e*_ (mg/g)	*R*	*α* (min^−1^)	*β* (mg/g)	*R*
25	0.116	8.199	0.954	0.009	8.6	0.983	19.75	0.903	0.941
35	0.230	8.909	0.961	0.012	8.86	0.988	9.73	0.537	0.944
50	0.581	8.703	0.967	0.038	9.75	0.985	3.596	0.658	0.940

**Table 2:** Kinetic parameters of lead sorption by calcium alginate beads at different temperatures.

Temperature (°C)	Pseudo-first-order (Lagergren)			Pseudo-second-order			Elovich		
	*K*_*L*_ (min^−1^)	*q*_*e*_ (mg/g)	*R* ^2^	*K*_2_ (min^−1^)	*q*_*e*_ (mg/g)	*R* ^2^	*α* (min^−1^)	*β* (mg/g)	*R* ^2^
25	0.092	12.434	0.918	0.009	13.663	0.992	22.711	0.582	0.973
35	21.279	15.595	0.918	0.031	15.716	0.998	9.261	1.767	0.971
50	20.231	15.921	0.919	0.057	15.921	0.999	4.429	3.307	0.975

**Table 3:** Isotherm model parameters and correlation coefficients (*R*) for cadmium biosorption by CAB.

*T* (°C)	Langmuir			Freundlich			Langmuir-Freundlich			
	*q*_0_ (mg/g)	*a*_*L*_ (L/mg)	*R*	*K*_*F*_ (mg/g)(mg/L)^1/*n*^	*n*	*R*	*K*_*LF*_ (mg/g)(mg/L)^1/*n*^	*a*_*LF*_ (mg/L)	*n*	*R*
25	27.439	0.384	0.969	8.883	2.76	0.991	8.953	0.003	0.36	0.971
35	32.935	0.477	0.977	11.600	2.86	0.995	12.734	0.004	0.41	0.975
50	39.317	0.667	0.985	16.103	3.90	0.991	25.959	0.063	0.95	0.985

**Table 4:** Isotherm model parameters and correlation coefficients (*R*) for lead biosorption by CAB.

*T* (°C)	Langmuir			Freundlich			Langmuir-Freundlich			
	*q*_0_ (mg/g)	*a*_*L*_ (L/mg)	*R*	*K*_*F*_ (mg/g)(mg/L)^1/*n*^	*n*	*R*	*K*_*LF*_ (mg/g)(mg/L)^1/*n*^	*a*_*LF*_ (mg/L)	*n*	*R*
25	150.4	0.021	0.978	23.887	2.45	0.968	1.836	0.008	0.23	0.991
35	283.3	0.229	0.917	98.087	4.45	0.977	4.826	0.004	0.42	0.989
50	316.1	0.051	0.968	68.948	3.13	0.985	6.581	0.002	0.67	0.986

**Table 5:** Dubinin-Radushkevich parameters for cadmium and lead sorption by CAB.

Parameter	Cd			Pb		
	25°C	35°C	50°C	25°C	35°C	50°C
*X*_*m*_ (mg/g)	20.45	21.75	26.6	138.38	159.17	267.73
*K*_*m*_	0.4547	0.0824	0.0675	1.94	0.824	0.0475
*E* (kJ/mol)	2.96	6.96	7.69	1.43	2.2	9.17
*R* ^2^	0.951	0.912	0.901	0.961	0.903	0.902
